# *LAT1 (SLC7A5)* Overexpression in Negative Her2 Group of Breast Cancer: A Potential Therapy Target

**DOI:** 10.31557/APJCP.2020.21.5.1453

**Published:** 2020-05

**Authors:** Khaldon Bodoor, Rowida Almomani, Mohammad Alqudah, Yazan Haddad, Walaa Samouri

**Affiliations:** 1 *Department of Applied Biology, Jordan University of Science and Technology, Irbid, Jordan. *; 2 *Department of Medical Laboratory Sciences, Jordan University of Science and Technology, Irbid, Jordan. *; 3 *Department of Pathology, Jordan University of Science and Technology, Irbid, Jordan. *; 4 *Department of Chemistry and Biochemistry, Mendel University in Brno, Zemedelska 1, Brno, Czech Republic. *; 5 *Central European Institute of Technology, Brno University of Technology, Purkynova, Brno, Czech Republic. *

**Keywords:** Breast cancer, LAT1, SLC7A5, HER2, ER- PR

## Abstract

**Objective:**

HER2 negative carcinomas of the breast pose a challenge for treatment due to redundancies in potential drug targets and poor patient outcomes. Our aim was to investigate the role of L-type amino acid transporter – LAT1 as a potential prognosticator and a drug target.

**Methods:**

In this retrospective work, we have studied the expression of LAT1 in 145 breast cancer tissues via immunohistochemistry. Overall survival analysis was used to evaluate patient outcome in various groups of our cohort.

**Results:**

Positive* LAT1* expression was found in 27 (84.4%) luminal A subtype, 27 (64.3%) luminal B/triple positive subtype, 29 (82.9%) triple negative subtype, and 24 (66.7%) HER2-only positive subtype (p=0.1). Interestingly, negative correlation was found between* LAT1* and *HER2*; where positive expression of LAT1 was found in 56 (83.6%) cases in negative HER2 group and 51 (65.4%) cases from positive HER2 group (p=0.01). Unfortunately, we were unable to report significant survival differences when *LAT1* expression was studied in the negative HER2 group. Nevertheless, five incidents of mortality (out of 55) were reported in LAT1+/HER2- group compared to none in the LAT1-/HER2- group (N=11).

**Conclusion:**

Our findings of overexpression of LAT1 in negative HER2 group suggest a role of this protein as prognosticator and drug target in a challenging therapeutic cohort.

## Introduction

LAT1 (solute carrier family 7 member 5; SLC7A5) is a member of the L-type amino acid transporter family which has 12 transmembrane domains and is covalently associated with solute carrier protein SLC3A2, also known as CD98. LAT1 functions in the bi-directional transport of essential neutral amino acids in and out of cells (Fotiadis et al., 2013). It is known to be overexpressed in multiple types of solid tumors including breast cancer, lung cancer, urogenital cancers, pancreatic cancer, gastrointestinal cancers, head and neck cancers, among others (Hafliger and Charles, 2019). The amino acid transporter LAT1 has recently attracted great attention for its role in breast cancer proliferation and survival (El Ansari et al., 2018a). Breast cancer molecular subtypes, i.e., based on the estrogen receptor (ER), progesterone receptor (PR) and receptor tyrosine-protein kinase erbB-2 *(HER2*) expression profiles, have helped in unraveling the heterogeneity nature of the disease. In particular, HER2 positive groups (e.g., Luminal A and luminal B subtypes) usually confer good patient outcome compared to the rest. Recent findings suggest potential role of metabolic programming of amino acid transporters including LAT1 in breast cancer heterogeneity (Cha et al., 2018; El Ansari et al., 2018c). Until now, the regulation of *LAT1 *expression is not well understood, and many transcriptional regulators have been connected with *LAT1* overexpression with no common denominator; e.g., c-Myc, hypoxia-inducible factor (HIF2α), and regulators of glucose transporters (Scalise et al., 2018).

RNA interference studies have shown that downregulation of *LAT1* expression results in growth inhibition in different cancer cell lines. Furthermore, inhibitors of LAT1 are currently being tested in preclinical studies in several types of cancer (Hafliger and Charles, 2019).

Herein, we constructed TMA of 145 samples of breast cancer subtypes to investigate the expression level of *LAT1* by immunohistochemistry and its correlation with different clinicopathological parameters.

## Materials and Methods

*Breast cancer samples*

This study was approved by Faculty of Medicine Research Ethics Committee at Jordan University of Science and Technology (Irbid, Jordan). A retrospective analysis of the pathological records from the department of pathology at King Abdullah University Hospital (Irbid, Jordan) identified 145 female patients diagnosed with breast cancer who underwent radical mastectomy for the tumor or axillary lymph node resection between the years 2007 and 2019. 

*TMA and Immunohistochemistry*

Tissue microarray (TMA) was constructed from archived paraffin-embedded breast carcinoma tissue blocks using the TMA Master II instrument (3DHISTECH Ltd., Budapest, Hungary). All TMA tissue blocks were sectioned at 4 μm thickness and collected on Superfrost plus glass slides for processing by immunohistochemistry. For immunohistochemistry, the BenchMark ULTRA system (Roche Diagnostics, Risch-Rotkreuz, Switzerland) was used to process the TMA slides for immunohistochemistry as previously described (Bodoor et al., 2018). Rabbit polyclonal antibody against LAT1 (SLC7A5) (Thermo Fisher Scientific, Waltham, MA, USA) at 1:100 dilution was used. The slides were scored as described previously (Bodoor et al., 2018) whereas slides with 1-33% expression were scored as weak and were considered negative in statistical analysis; slides with 34-66% expression were scored as moderate and were considered positive; and slides with 67-100% expression were scored as strong and were also considered as positive in statistical analysis. *ER*, *PR* and *HER2* expression was taken from the archived records. Tumor volume was calculated differently according to available data for dimensions (For one dimension x: volume = 4/3*π*(x/^2^)^3^; for two dimensions x and y whereas x<y: volume = x^2^*y; and for three dimensions x, y and z: volume = x*y*z). 

*Statistical Analysis*

Pearson *χ*^2^ test of independence was used to compare clinical and pathological characteristics with* LAT1* expression. One-sided Fisher’s exact test was considered more reliable for 2×2 crosstabs and thus was reported instead of *χ*^2^ test where appropriate. The Kaplan-Meier survival curves were used to represent the overall survival distributions, defined as the period from time of diagnosis to death from any cause or the last contact. The difference in overall survival according to *LAT1* expression and clinical and pathological characteristics were analyzed using the log rank test (Mantel, 1966) giving equal weights to individuals at all-time intervals. Data spreadsheet was prepared in Microsoft Excel 2013 (Microsoft, Redmond, WA, USA) and further analysis was done using IBM SPSS Statistics for Windows, Version 21 (IBM Corp, Armonk, NY, USA). p-value ≤0.05 was considered significant.

**Figure 1:**
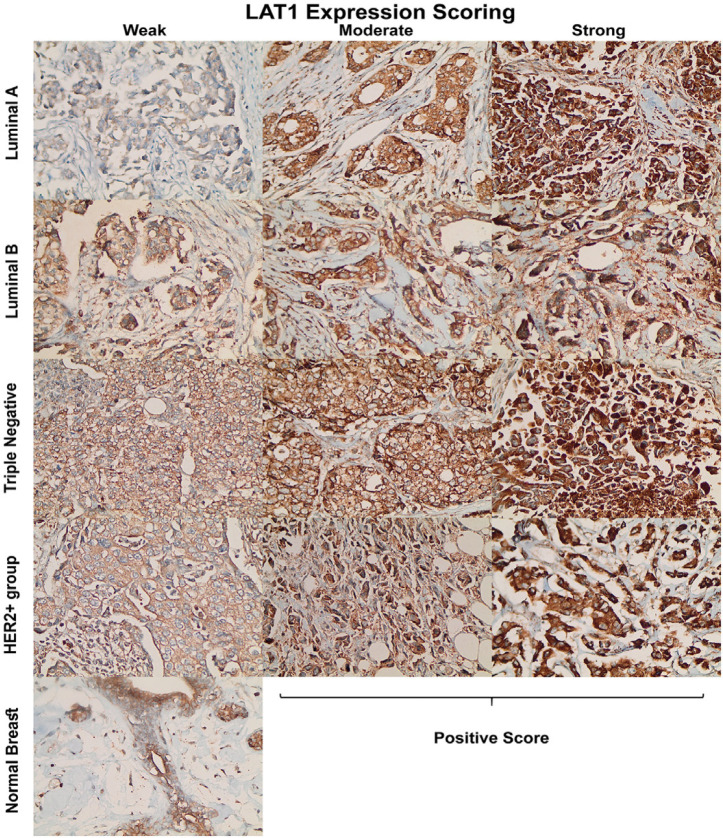
Representative Immunohistochemistry Images for *LAT1* Expression and Scoring at 400× Magnification

**Table 1:** Histopathological, Clinical Characteristics and *LAT1* Expression of 145 Breast Cancer Patients. Significant correlation was found between positive LAT1 expression and negative HER2 expression which represents 56 patients of all studied cases

Characteristic	Group	*LAT1* Expression		Total	p-value
		Negative	Positive	N=145	
		N=38 (%)	N=107 (%)		
Age	≤50 years	20 (29.4)	48 (70.6)	68	0.3
	>50 years	18 (23.4)	59 (76.6)	77	
Molecular subtypes	Luminal A	5 (15.6)	27 (84.4)	32	0.1
	Luminal B (Triple Positive)	15 (35.7)	27 (64.3)	42	
	Triple Negative	6 (17.1)	29 (82.9)	35	
	HER2-Only Positive	12 (33.3)	24 (66.7)	36	
*ER/PR* Expression	ER-/PR-	18 (25.4)	53 (74.6)	71	0.5
	ER+/PR+	20 (27.0)	54 (73.0)	74	
*HER2 *Expression	Negative	11 (16.4)	56 (83.6)	67	0.01*
	Positive	27 (34.6)	51 (65.4)	78	
Histological type	Invasive Ductal Carcinoma	32 (26.2)	90 (73.8)	122	0.6
	Medullary Carcinoma	3 (42.9)	4 (57.1)	7	
	Metaplastic Carcinoma	1 (25.0)	3 (75.0)	4	
	Basal or Basal-like Carcinoma	1 (12.5)	7 (87.5)	8	
	Micropapillary Carcinoma	0 (0)	3 (100)	3	
Ductal Carcinoma in situ (DCIS)	Absent	6 (20.7)	23 (79.3)	29	0.3
	Present	29 (29.0)	71 (71.0)	100	
Axillary Lymph Nodes	Negative	8 (22.2)	28 (77.8)	36	0.3
	Positive	27 (27.8)	70 (72.2)	97	
Lymph node Vascular Invasion	Absent	8 (25.8)	23 (74.2)	31	0.5
	Present	23 (27.7)	60 (72.3)	83	
Tumor volume	<10 cm^3^	9 (25.7)	26 (74.3)	35	0.6
	10–30 cm^3^	12 (30.0)	28 (70.0)	40	
	>30 cm^3^	13 (21.3)	48 (78.7)	61	
Tumor size	T1	4 (50.0)	4 (50.0)	8	0.08
	T2	19 (24.4)	59 (75.6)	78	
	T3	8 (19.0)	34 (81.0)	42	
	T4	7 (46.7)	8 (53.3)	15	
Lymph node status	N0	9 (24.3)	28 (75.7)	37	0.2
	N1	11 (33.3)	22 (66.7)	33	
	N2	4 (14.3)	24 (85.7)	28	
	N3	12 (35.3)	22 (64.7)	34	
Distant metastasis	M0	20 (26.0)	57 (74.0)	77	0.9
	M1	14 (27.5)	37 (72.5)	51	
Stage	I	2 (50.0)	2 (50.0)	4	0.7
	II	11 (26.8)	30 (73.2)	41	
	III	6 (21.4)	22 (78.6)	28	
	IV	14 (27.5)	37 (72.5)	51	
Histological grade	G1	2 (33.3)	4 (66.7)	6	0.8
	G2	8 (22.9)	27 (77.1)	35	
	G3	28 (27.2)	75 (72.8)	103	
Family History	No	13 (26.0)	37 (74.0)	50	0.3
	Yes	7 (18.9)	30 (81.1)	37	

* *p*-value≤0.05 was considered significant.

**Table 2:** Overall Survival Analysis of Differences between the Pathophysiological Groups. Increase in tumor size, lymph node status, metastasis, and lymph node vascular invasion were significantly correlated with poor patient outcome

Characteristic	Group	N	Deaths	Mean Survival±SE (years)	*p*-value
Age	≤50 years	62	5	10.0±0.4	0.6
	>50 years	74	8	12.5±0.8	
Molecular subtypes	Luminal A	31	3	9.1±0.4	1
	Luminal B (Triple Positive)	41	5	9.8±0.5	
	Triple Negative	35	3	9.9±0.6	
	HER2-Only Positive	29	2	13.7±0.9	
ER/PR Expression	Negative	64	5	13.5±0.6	0.9
	Positive	72	8	9.8±0.4	
HER2 Expression	Negative	66	6	9.7±0.5	0.9
	Positive	70	7	13.2±0.7	
Histological type	Invasive Ductal Carcinoma	114	12	N/A	0.8
	Medullary Carcinoma	6	0		
	Metaplastic Carcinoma	4	0		
	Basal or Basal-like Carcinoma	8	1		
	Micropapillary Carcinoma	3	0		
Ductal Carcinoma in situ (DCIS)	Absent	29	5	8.1±0.6	0.09
	Present	94	7	11.8±0.4	
Axillary Lymph Nodes	Negative	36	2	13.9±0.8	0.08
	Positive	89	11	10.7±0.7	
Lymph node Vascular Invasion	Absent	28	0	N/A	0.01*
	Present	78	11		
Tumor volume	<10 cm^3^	34	1	14.0±0.9	0.2
	10–30 cm^3^	38	5	9.6±0.5	
	>30 cm^3^	56	6	11.5±0.6	
Tumor size	T1	8	0	N/A	0.2
	T2	72	6		
	T3	40	6		
	T4	14	1		
Lymph node status	N0	37	2	N/A	0.001*
	N1	30	0		
	N2	26	3		
	N3	31	8		
Metastasis	None	77	1	10.9±0.1	<0.001*
	With metastasis	50	12	7.3±0.7	
Stage	I	4	0	N/A	<0.001*
	II	41	0		
	III	28	1		
	IV	50	12		
Histological grade	G1	6	0	N/A	0.2
	G2	34	2		
	G3	96	11		
Family History	No	49	6	9.7±0.5	0.3
	Yes	37	3	10.1±0.5	

SE, standard error; N/A, not applicable; *, p-value≤0.05 was considered significant; Statistical analysis was done using Logrank test.

**Figure 2:**
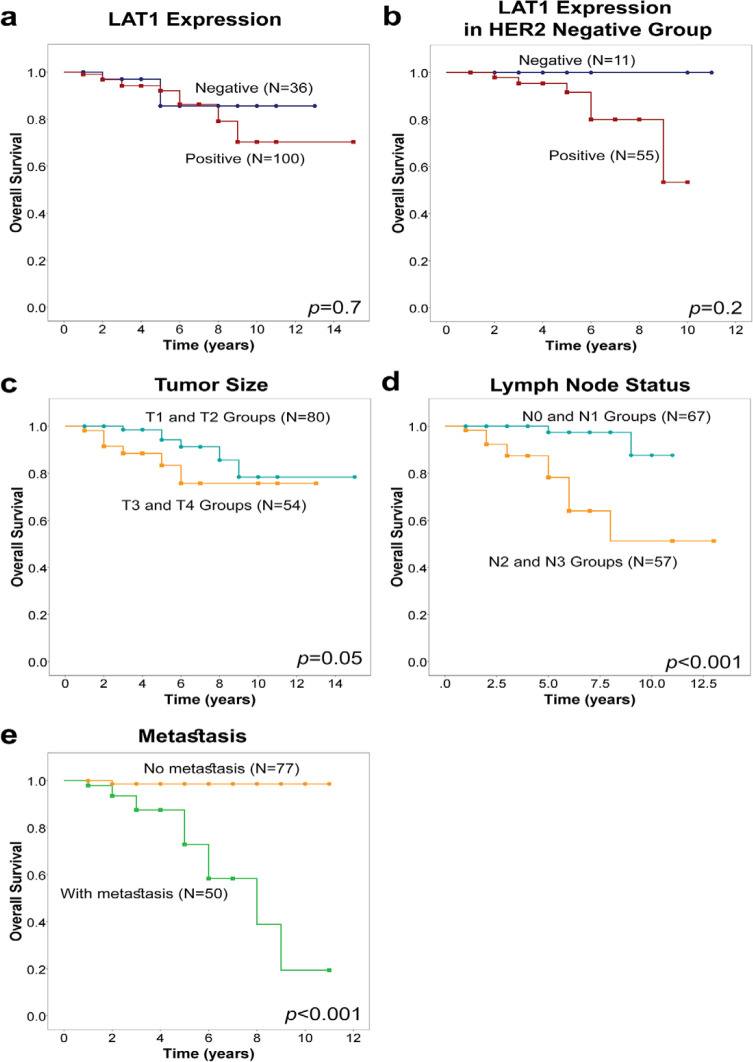
Kaplan-Meier Curves for Overall Survival among Breast Cancer Patients. (a) Overall survival according to LAT1 expression. (b) Overall survival according to LAT1 expression in HER2 negative group. (c) Overall survival according to tumor size. (d) Overall survival according to lymph node status. (e) Overall survival according to metastasis. Overall survival time was defined until event of death from any cause (drop in curve) or last contact (censored cases are shown as circles and squares). p-value≤0.05 was considered significant

## Results

Of the 145 patients, 68 women were age 50 years or less while 77 were older than 50 years old. The mean age ± standard deviation was 51.7±11.0 (range 30–82 years). Samples of four subtypes were included in the study; namely, 32 luminal A (ER+/PR+/HER2-), 42 luminal B (ER+/PR+/HER2+), 35 triple negative (ER-/PR-/HER2-), and 36 HER2-only positive (ER-/PR-/HER2+). *LAT1* expression was scored according to percentage of positive cells (as described in the methods section), and it was categorized into “negative” for both null and weak; and “positive” for both moderate and strong (Figure 1). Positive *LAT1* expression was found in 27 (84.4%) luminal A subtype, 27 (64.3%) luminal B/triple positive subtype, 29 (82.9%) triple negative subtype, and 24 (66.7%) HER2-only positive subtypes (Table 1, p=0.1). With exception of *HER2* expression groups (*χ*^2^ test p=0.01), no significant correlation was found between *LAT1* expression and any of the other clinicopathological characteristics (Table 1). As we have mentioned before, a negative correlation was found between LAT1 and HER2 (Table 1, p=0.01), and we have decided to investigate this further. Positive expression of *LAT1* was found in 56 (83.6%) cases from the negative HER2 group and 51 (65.4%) cases from the positive HER2 group. In contrast, survival analysis did not show significant difference between negative and positive *LAT1* expression (Figure 2a, p=0.7). Furthermore, we investigated overall survival according to *LAT1* expression in the negative HER2 group (Figure 2b). Unfortunately, due to the low number of LAT1-/HER2- samples, we were unable to report significant survival differences when *LAT1* expression was studied in this cohort. Nevertheless, five incidents of mortality (out of 55) were reported in LAT1+/HER2- group compared to none in the LAT1-/HER2- group (N=11). 

In contrast, survival analysis (Table 2) showed significant role of some of the histopathological characteristics particularly tumor size (Figure 2c, p=0.05), lymph node status (Figure 2d, p<0.001) metastasis (Figure 2e, p<0.001) and stage (Table 2, p=0.004).

Overall, these findings highlight high expression of *LAT1* in negative HER2 group, and also show histopathological characteristics (particularly metastasis) as the main determinants of patients’ outcome. 

## Discussion

Previous genomic and TMA studies of large breast cancer cohorts have shown that LAT1 (SLC7A5) is a prognostic indicator of poor patient outcome in luminal B subtype only (Ring et al., 2006; El Ansari et al., 2018b; El-Ansari et al., 2019; Sevigny et al., 2019). In contrast, Furuya et al., (2012) have shown* LAT1 *expression is higher in HER2+ and triple negative groups when compared to the luminal A and B subtypes and is associated with poor outcome in triple negative patients. Additionally, Liang et al., (2011) demonstrated that high grade and stage breast cancer tissues (HER2+ and triple negative groups) express higher levels of *LAT1*. Here we identified relatively high expression of *LAT1 *in all subtypes and particularly higher expression in the negative *HER2* groups that are usually more difficult to treat. Although the LAT1 positive group displayed more fatalities, however, that finding was proportional to the number of patients. Here, survival analysis demonstrated clear role of histopathological features related to cancer progression and metastasis but not protein expression (Table 2). Nevertheless, *LAT1* high expression in breast cancer provides a new opportunity as a therapeutic target particularly in subtypes that are difficult to treat. Indeed, many strategies targeting LAT1 are under development including potent LAT1 inhibitors (Napolitano et al., 2017; Singh et al., 2018). Alternatively, LAT1 is known to bind cholesterol in the plasma membrane, which can be applied as potential modulation approach to alter its activity (Dickens et al., 2017). LAT1 fits several criteria that are required for identifying cell surface protein targets for use in immunotherapy (Scott et al., 2012) and nanomedicine-based drug delivery (Haddad et al., 2017), such as high expression, accessibility and also identified crystal 3D structure (Yan et al., 2019). Recently, Häfliger and Charles (2019) reviewed current LAT1-targeting therapies particularly the ones in preclinical stage. Our findings support further investigations of this protein for use in treatment of breast cancer, particularly in cohorts that lack any druggable targets.

## Author Contribuiions

WS and MA performed TMA and immune-histochemical analysis. YH performed statistical analysis. KB,YH and RA wrote the manuscript. KB was the principal investigator of this work.
